# BMP1 is not required for lung fibrosis in mice

**DOI:** 10.1038/s41598-022-09557-3

**Published:** 2022-03-31

**Authors:** Hsiao-Yen Ma, Elsa-Noah N’Diaye, Patrick Caplazi, Zhiyu Huang, Alexander Arlantico, Surinder Jeet, Aaron Wong, Hans D. Brightbill, Qingling Li, Weng Ruth Wong, Wendy Sandoval, Lucinda Tam, Robert Newman, Merone Roose-Girma, Ning Ding

**Affiliations:** 1 Department of Discovery Immunology, Genentech, South San Francisco, CA USA; 2 Department of Pathology, Genentech, South San Francisco, CA USA; 3 Department of Translational Immunology, Genentech, South San Francisco, CA USA; 4 Department of Microchemistry, Proteomics and Lipidomics, Genentech, South San Francisco, CA USA; 5 Department of Molecular Biology, Genentech, South San Francisco, CA USA

**Keywords:** Drug discovery, Diseases, Molecular medicine

## Abstract

Bone morphogenetic protein 1 (BMP1) belongs to the astacin/BMP1/tolloid-like family of zinc metalloproteinases, which play a fundamental role in the development and formation of extracellular matrix (ECM). BMP1 mediates the cleavage of carboxyl terminal (C-term) propeptides from procollagens, a crucial step in fibrillar collagen fiber formation. Blocking BMP1 by small molecule or antibody inhibitors has been linked to anti-fibrotic activity in the preclinical models of skin, kidney and liver fibrosis. Therefore, we reason that BMP1 may be important for the pathogenesis of lung fibrosis and BMP1 could be a potential therapeutic target for progressive fibrotic disease such as idiopathic pulmonary fibrosis (IPF). Here, we observed the increased expression of BMP1 in both human IPF lungs and mouse fibrotic lungs induced by bleomycin. Furthermore, we developed an inducible *Bmp1* conditional knockout (cKO) mouse strain. We found that Bmp1 deletion does not protect mice from lung fibrosis triggered by bleomycin. Moreover, we found no significant impact of BMP1 deficiency upon C-term propeptide of type I procollagen (CICP) production in the fibrotic mouse lungs. Based on these results, we propose that BMP1 is not required for lung fibrosis in mice and BMP1 may not be considered a candidate therapeutic target for IPF.

## Introduction

Characterized by excessive deposition of excessive collagen matrix proteins, idiopathic pulmonary fibrosis (IPF) is a progressive fibrotic disease that is associated with high morbidity and mortality^1^. IPF is the pathological result of repetitive microinjuries to lung airway and alveolar epithelium, which provoke the accumulation of myofibroblasts and the deposition of collagen fibrils in the interstitial regions^2,3^. As the common and final step for IPF as well as other fibrotic diseases, excessive collagen fibril formation and deposition lead to loss of lung function and, ultimately, death^4,5^. Hence, the mediators of this critical biological process may represent appealing therapeutic targets for IPF^4,5^.

Collagen fibril formation is a complex process involving multiple posttranslational modifications^6,7^. One of the central steps in this process is the proteolytic processing of procollagens^6,7^. Once synthesized within cells, monomeric collagen chains are packaged and secreted into triple-helix procollagen molecules with amino (N-term) and carboxyl terminal (C-term) propeptides, which need to be cleaved to enable the subsequent formation of supramolecular collagen fibrils in the extracellular matrix (ECM). At this step, C-term propeptide removal seems to be more important than N-term processing in their ability to trigger collagen fibrillogenesis^8–11^.

Multiple proteinases have been discovered to exhibit enzymatic activity to cleave C-term propeptides from procollagens within the past few decades^12–16^. Among them, the bone morphogenetic protein 1 (BMP1) appears to be the main enzyme responsible for the fibrillar procollagen maturation, particularly type I procollagen^14,15,17,18^. Human genetics studies identified several loss-of-function *BMP1* mutations associated with Osteogenesis Imperfecta (OI), an inheritable brittle bone disease largely due to autosomal dominant mutations in *COL1* genes^19–28^, supporting a critical role of BMP1 in type I procollagen maturation and processing during human bone development. It has also been shown that inhibition of BMP1 by small molecules or antibody reduces skin^29,30^, kidney^31,32^ and liver fibrosis^33^ in the animal models. In line with these reports, we have recently shown that genetic deletion or pharmacological inhibition of BMP1 abrogates type I C-terminal procollagen propeptide (CICP) production and collagen deposition from primary lung fibroblasts^34^.

Considering the important role of BMP1 in procollagen processing, maturation and deposition, we hypothesized that BMP1 may be a critical determinant of lung fibrosis and could be a potential therapeutic target for progressive fibrotic diseases such as IPF. Upon analyses of fibrotic lung samples from human and mouse, we found BMP1 expression is increased in fibrotic lesions. Next, we developed a mouse model where efficient deletion of both *Bmp1* alleles can induced by tamoxifen treatment. However, when these *Bmp1* conditional knockout (cKO) mice were tested in the bleomycin model of lung fibrosis, we observed that BMP1 deficiency does not prevent lung fibrosis in mice. Further examination of CICP production also revealed that BMP1 is not necessary for type I procollagen C-term processing and maturation during lung fibrosis. Taken together, we provided the first genetic evidence that BMP1 is not required for the development of lung fibrosis in mice, thus arguing against the drug target candidacy of BMP1 for IPF.

## Results

### BMP1 expression is increased in IPF lungs

**Figure 1:**
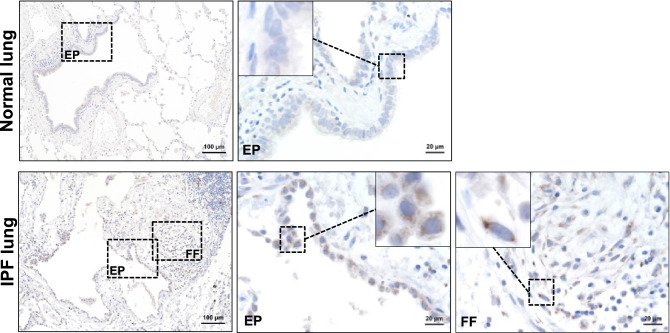
BMP1 expression is increased in human IPF lungs. Representative images of BMP1 immunochemistry staining in human IPF and normal lung specimens. *EP* epithelial cells, *FF* fibroblastic foci.

We first assessed the protein level of BMP1 by immunohistochemical staining in lung samples collected from normal control and IPF patients. We only observed a minimal signal of BMP1 protein in normal lung. On the contrary, the immunoreactivity of BMP1 was strongly detected in IPF lungs. Specifically, BMP1 protein appears to be enriched in the fibroblastic foci (FF), a histological feature of IPF, and the epithelium lining (Fig. 1).

### BMP1 expression is increased in the bleomycin model of lung fibrosis

**Figure 2:**
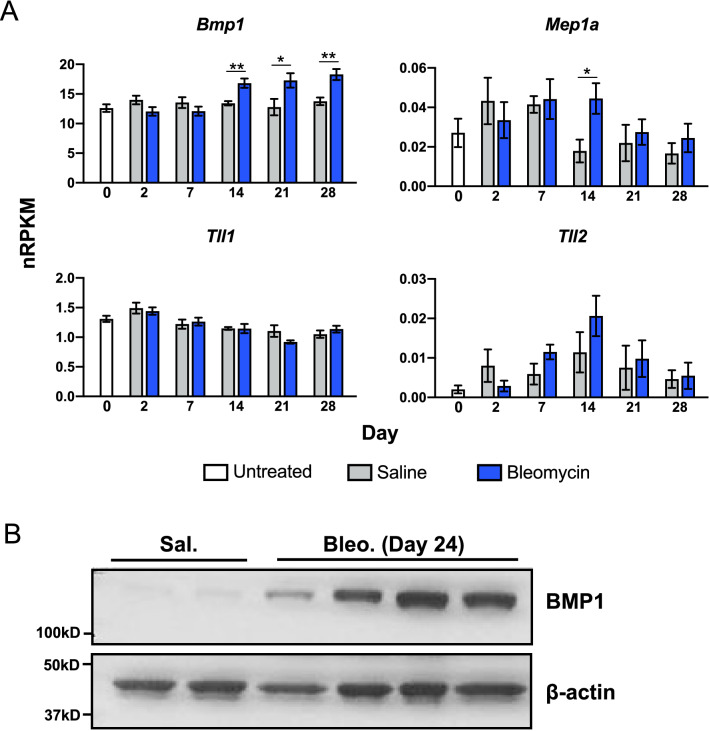
BMP1 expression is increased in the bleomycin model. (**A**) mRNA levels of *Bmp1*, *Tll1*, *Tll2* and *Mep1a* in mouse lungs on indicated days (0, 2, 4, 7, 14, 21 and 28, n = 5–7) after bleomycin challenge. *p < 0.05, **p < 0.01. Data represents mean ± S.E.M. p-value is calculated using one-way ANOVA. (**B**) Representative western blot of BMP1 (mTLD, the longer spliced version of BMP1) in lung lysates after 24 days bleomycin challenge compare to saline group, each lane represents single biological sample. β-actin is a loading control.

To complement our observation in human samples, we sought to determine whether BMP1 expression is increased in the mouse model of lung fibrosis. Intratracheal (IT) instillation of bleomycin model has been widely used as an animal model to induce experimental lung fibrosis. Mechanistically, bleomycin damages lung epithelium, which results in an immune response and fibroblasts activation, differentiation and ECM deposition^35^. Through analysis of a previously published transcriptomics data in the bleomycin model^36^, we found that *Bmp1* expression was trending slightly lower, though not significant, in the bleomycin-challenged moue lungs at early time points (day 2 and 7) but was significantly increased at the advanced fibrotic stage of the model (day 14–28) (Fig. 2A). We also examined the expression of other procollagen C-term proteinases including tolloid-like 1 (*Tll1),* tolloid-like 2 (*Tll2*), meprin A (*Mep1a*) and meprin β (*Mep1b*) in the same dataset. The expression of these genes was not significantly increased in the bleomycin injured mouse lungs throughout the experiment except that *Mep1a* expression was significantly increased only at day 14 (Fig. 2A). *Mep1b* expression was below the detection limit (data not shown). Consistent to the results from transcriptomic analyses, we found that BMP1 (the longer spliced version of *Bmp1,* also named as mTLD, ~ 120KD) protein level was dramatically elevated in mouse lungs 24 days after the challenge of the bleomycin while the shorter spliced version of BMP1 (~ 90KD) appears to be below the detection limit (Fig. 2B). These results thus suggest that the bleomycin model may be suitable to investigate the in vivo role of BMP1 in lung fibrosis.

### Inducible and efficient deletion of *Bmp1* alleles in *Bmp1* cKO mice

**Figure 3:**
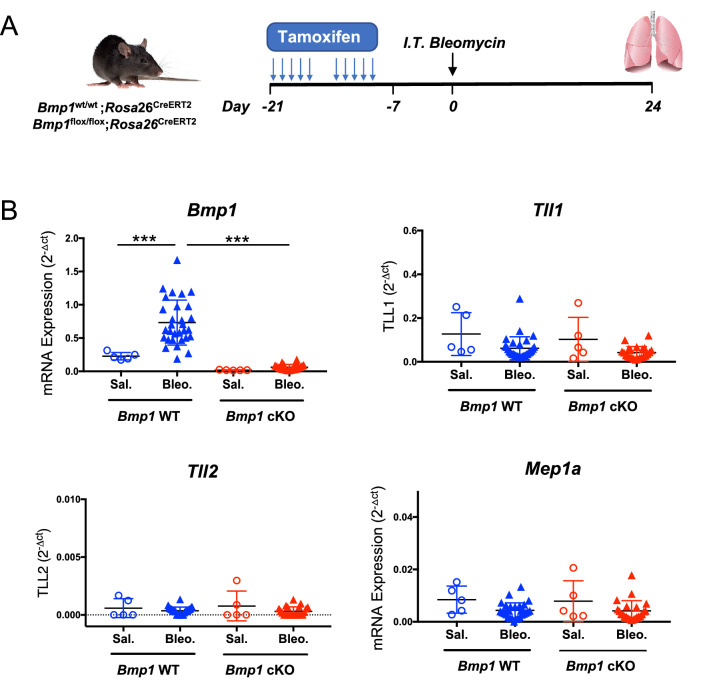
Inducible and efficient deletion of *Bmp1* in the bleomycin model. (**A**) Schematic regime of tamoxifen-induced *Bmp1* deletion following by intratracheal bleomycin challenge. Tissue harvesting on day 24 after bleomycin challenge. (**B**) mRNA expression of *Bmp1*, *Tll1*, *Tll2* and *Mep1a*. *Bmp1* WT: Saline n = 5, bleomycin n = 30; *Bmp1* cKO: saline n = 5, bleomycin n = 21. ***p < 0.001. Data represents mean ± SD. p-value is calculated using one-way ANOVA.

*Bmp1* constitutional knockout mice die soon after birth from the failure of ventral body wall closure due to abnormal collagen fibrillogenesis^31^. To bypass this postnatal lethality, we generated mice homozygous for the floxed (cKO) or wild-type (WT) *Bmp1* alleles and hemizygous for a Cre-ERT2 fusion transgene driven by the human Rosa26 promoter. Thus, both *Bmp1* alleles can be deleted when these cKO mice are treated with tamoxifen. In order to confirm the efficient deletion of Bmp1 alleles, we administered tamoxifen to *Bmp1* cKO and WT mice through intraperitoneal (IP) injection a week prior to IT injection of bleomycin or saline (Fig. 3A). Lung tissues were then harvested for gene expression analyses on day 24 after the initial bleomycin challenge (Fig. 3A). The data showed that tamoxifen induced more than 90% loss of *Bmp1* transcript in lungs in the presence or absence of bleomycin when compared to WT controls (Fig. 3B). In addition, we observed that *Tll1*, *Tll2* and *Mep1a* expression was not significantly affected by *Bmp1* deletion, suggesting that the loss of BMP1 is unlikely compensated by the increased expression of other C-proteinases as examined.

### *Bmp1* cKO mice are not protected from bleomycin-induced lung fibrosis

**Figure 4:**
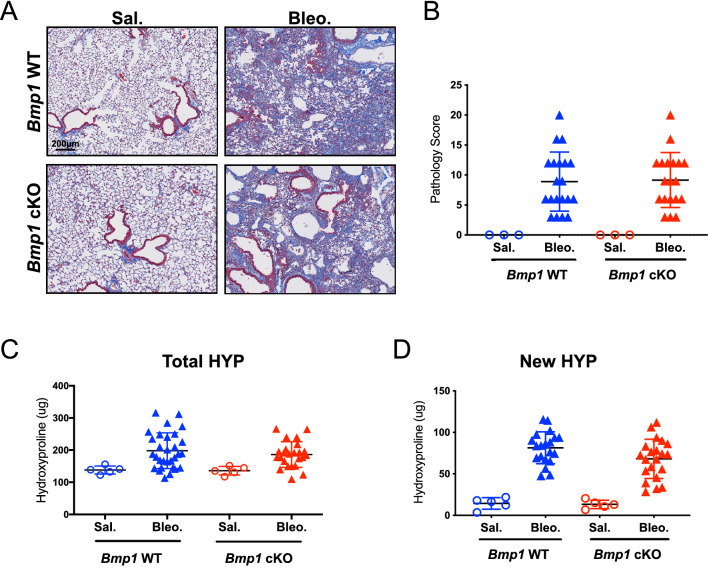
*Bmp1* cKO mice are not protective against bleomycin-induced lung fibrosis. Representative images of trichrome histology staining (**A**) in lung tissues with quantification of fibrosis lesion (**B**). Quantification of fibrosis by total hydroxyproline (**C**) and newly synthesized hydroxyproline (**D**). *Bmp1* WT: Saline n = 5, bleomycin n = 30; *Bmp1* cKO: saline n = 5, bleomycin n = 21. Data represents mean ± SD.

**Figure 5:**
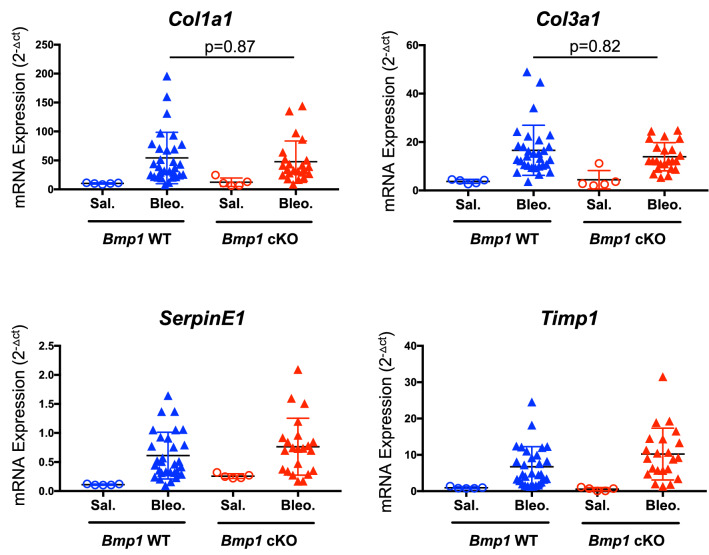
*Bmp1* deletion has no effects on pro-fibrotic gene expression. mRNA expression of selective pro-fibrotic genes from lung tissue. *Bmp1* WT: Saline n = 5, bleomycin n = 30; *Bmp1* cKO: saline n = 5, bleomycin n = 21. Data represents mean ± SD. p-value is calculated using one-way ANOVA.

Next, we investigated whether inducible deletion of *Bmp1* protects mice from the development of lung fibrosis in the bleomycin model. To this end, we first preformed the histological analysis to assess the severity of fibrotic lesions in the lungs of *Bmp1* cKO and WT mice. The data indicate there is no difference of the severity of fibrotic lesions in the lungs between *Bmp1* WT and cKO mice (Fig. 4A,B). Furthermore, using hydroxyproline assay, we quantified the overall collagen deposition in lungs and found no significant impact of *Bmp1* deletion on total hydroxyproline level in lungs (Fig. 4C,D). We also measured the newly synthesized hydroxyproline and found that *Bmp1* deletion does not affect this readout either, suggesting that BMP1 is unlikely to contribute to active collagen synthesis and production. Consistent to the histology and hydroxyproline data, we observed that the representative pro-fibrotic gene (*Col1a1*, *Col3a1*, *Serpine1* and *Timp1*) expression was not significantly impacted by *Bmp1* deletion (Fig. 5). Collectively, these results suggest that BMP1 does not contribute to the pathogenesis of lung fibrosis in the bleomycin model.

### *Bmp1* deletion does not reduce CICP production during bleomycin-induced lung fibrosis

**Figure 6:**
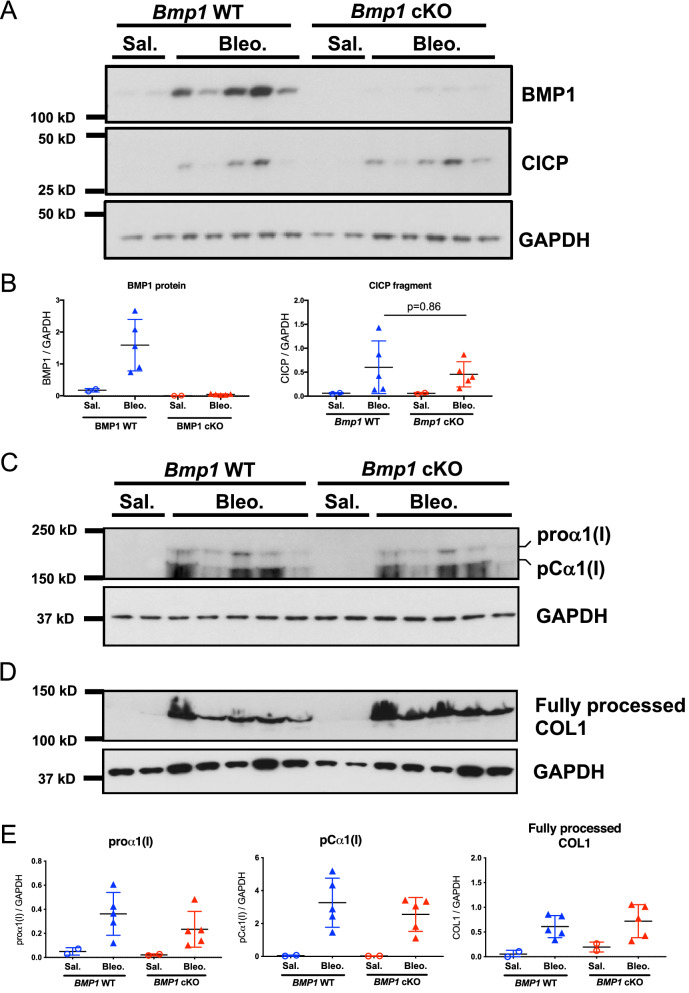
*Bmp1* deletion does not reduce CICP production during bleomycin-induced lung fibrosis. (**A**) Equal amounts of total proteins were subject to western blot for the detection of BMP1 and CICP in lung tissues. GAPDH was used as a loading control. (**B**) Quantification of relative protein expression of BMP1 and CICP in lung tissues by normalizing with GAPDH. (**C**,**D**) Representative western blot of proα1(I), pCα1(I) and fully processed COL1 in lung lysates, each lane represents single biological sample. (**E**) Quantification of relative protein expression of proα1(I), pCα1(I) and fully processed COL1 normalized by GAPDH. Data represents mean ± SD. p-value is calculated using one-way ANOVA.

The lack of anti-fibrotic effects in *Bmp1* cKO mice prompted us to explore whether BMP1 modulates procollagen, particularly type I procollagen, C-term processing during the development of lung fibrosis in the bleomycin model. We performed western blot (WB) in lung extracts from a satellite group of *Bmp1* WT and cKO mice treated with bleomycin or saline to assess the proα1(I), pCα1(I), fully processed COL1 and CICP production. While the BMP1 protein level appears to be efficiently depleted in the lungs of cKO mice, we found no significant change of proα1(I), pCα1(I), fully processed COL1 and CICP levels upon BMP1 depletion while their production were substantially increase in the bleomycin injured lungs (Fig. 6A–E). Therefore, these results suggest that BMP1 is not necessary for type I procollagen C-term processing and maturation during lung fibrosis in mice, which may explain the redundant role of BMP1 in lung fibrosis.

## Discussion

As IPF has the worst prognosis among all fibrotic diseases and most IPF patients survive less than 3 years from diagnosis, this fatal and progressive fibrotic lung disease placed a huge burden on the global public health system^1–3^. However, very few experimental therapies exhibited clinical benefits in human patients and the current standard of care medications, pirfenidone and nintedanib, only slow down the decline of lung function without significantly improving patient survival^37,38^. New treatment options are therefore urgently needed to prevent or even reverse the progression of IPF toward disease mortality^4,5^.

Since its discovery as the first C-term proteinase in early 90s^14,15^, BMP1 has long been proposed to be an appealing therapeutic target for progressive fibrotic diseases^39^. Within the past few decades, several potent hydroxamic acid-containing small molecule BMP1 inhibitors have been reported, but no advancement to clinical trial has been documented^29,40–42^ possibly due to poor selectivity and/or poor metabolic stability^43^. More recently, BMP1 antibody inhibitors have been reported^31,34^ and one of them exhibited anti-fibrotic efficacy in preclinical models of kidney and liver fibrosis^31,33^. However, to the best of our knowledge, there are no genetics studies that have ever validated the role of BMP1 in the pathogenies of tissue fibrosis.

In this end, we developed a *Bmp1* cKO mouse model where both *Bmp1* alleles can be deleted in the adulthood of the animals. This unique mouse model allows us to fully employ a genetic approach to determine the contribution of BMP1 to lung fibrosis in animals. To our surprise, we found that BMP1 is clearly not required for lung fibrosis in the bleomycin model and BMP1 does not contribute to type I procollagen C-term processing. These findings not only suggest that BMP1 may not be a suitable drug target for progressive lung fibrotic diseases but also indicate that BMP1 is not the major C-term proteinase for procollagen in this particular model. These results are somewhat contradictory, but not mutually exclusive, with previous reports of BMP1’s dominant role in collagen deposition or procollagen C-term processing using BMP1 inhibitors and in vitro cell culture system^17,18,29–31,33,34^. Herein, we suggest that there could be three explanations to reconcile these discrepancies.

First of all, a few non-BMP1 proteinases are also able to cleave C-term propeptide of procollagen. For instance, due to the similar domain structures, TLL1 and TLL2 were identified as BMP1-like proteinases and all these BMP1/tolloid-like proteinases (BTPs) can process C-term procollagen efficiently^12,13^. MEP1A and MEP1B have also been identified as procollagen C-term proteinases^16^. Hence, the functional redundancy between these C-proteinases may well compensate the depletion of BMP1 in vivo. In support of this notion, genetic deletion of *Bmp1* or *Bmp1/Tll1* in mouse embryonic fibroblasts (MEFs) suggested that both BMP1 and TLL1 contribute to C-term procollagen processing^17^. On the other hand, as tissue fibrosis ensues from a pathological microenvironment, multiple cell types can be involved in this process. Thus, it is possible that proteinase activities from non-fibroblastic cell types may contribute to C-term processing of procollagens as well. Therefore, future studies using genetic models or unbiased biochemical analyses are needed to identify which proteinases are truly responsible for procollagen C-term processing and maturation during lung fibrosis in vivo.

In addition to the functional redundancy, we cannot ignore the possibility that BMP1 may play a tissue specific role in the pathogenesis of fibrosis. It is noteworthy that, while previous studies tested BMP1 inhibitors in the animal models of skin^29,30^, kidney^31,32^ and liver fibrosis^33^, the anti-fibrotic efficacy of BMP1 inhibitors in lung fibrosis models has not been documented. Thus, it is plausible that BMP1 may play a much more important role in skin, kidney and liver fibrosis than in lung fibrosis. This possibility can be further supported by human genetics data that the collagen abnormality caused by BMP1 loss-of-function mutations appears to be largely restricted to bone tissue^19–28^. Nonetheless, additional studies on *Bmp1* cKO mice in the fibrosis models of other tissues are mandatory to fully test this hypothesis.

Thirdly, as the selectivity for both small molecule and antibody BMP1 inhibitors has not been fully characterized, it may be reasonable to speculate that these molecules may hit targets beyond BMP1 in vivo, if the anti-fibrotic effects of these BMP1 inhibitors can be independently reproduced. Given the high sequence and structure similarity between BTP family members^44^, it is highly likely that BMP1 inhibitors may target the entire BTP family to achieve their anti-fibrotic effects. However, as the CICP level was not determined in the previous pharmacology studies in animal models, we cannot exclude another possibility that the anti-fibrotic effects of these inhibitors may, at least partially, come from non-BTP targets, which may be functionally relevant to tissue fibrosis.

Overall, our study provided the first genetic evidence that BMP1 is not required for lung fibrosis and BMP1 is not the major proteinase to cleave C-term of type I procollagen in the bleomycin model. Based on these findings, we thus propose that BMP1 should not be considered as a candidate therapeutic target for fibrotic lung diseases like IPF. More importantly, our study exemplifies the necessity of leveraging genetic models to validate drug targets when possible and suggests that the role of BMP1 in the pathological tissue fibrosis may need to be re-evaluated using the similar genetic approaches.

## Materials and methods

### Human samples

Explanted lung tissues were obtained from patients with a pathological diagnosis of usual interstitial pneumonia and a consensus clinical diagnosis of IPF assigned by multidisciplinary discussion and review of clinical materials. Written informed consent was obtained from all subjects and the study was approved by the University of California, San Francisco (UCSF) institutional review board. Human lungs not used by the Northern California Transplant Donor Network were used as controls; studies indicate that these lungs are physiologically and pathologically normal^45^. All procedures were carried out according to the Helsinki Declaration and its later amendments.

### Immunohistochemistry staining

4 μm sections of formalin-fixed and paraffin-embedded specimens were deparaffinated followed by antigen retrieval using Target Retrieval (Dako #S1700) and quenching endogenous peroxidase activity. The sections were subsequently blocked with Vector Avidin Biotin Blocking Kit, stained in PBS plus 10% rabbit serum and 3% BSA with anti-BMP1 (0.5 µg/ml, R&D, #AF1297) for lung sections and secondary biotinylated antibodies, incubated with Vectastain ABC Elite reagent and Pierce Metal Enhanced DAB solution and counterstained with Mayer's hematoxylin. Human sections were imaged with a 20 × Plan Apo DIC M objective (NA: 0.75, Nikon) on a Nikon Ti-Eclipse inverted microscope equipped with an Andor Neo scMOS camera (Andor, Oxford Instruments), a linear-encoded automated stage (Applied Scientific Instrumentation), and a SOLA LED light engine (Lumencor) all run by NIS Elements software (Nikon).

### Induced excision of Bmp1 in adult mice

All animal studies were conducted in accordance with the Guide for the Care and Use of Laboratory Animals, published by the National Institutes of Health (NIH) (NIH Publication 8523, revised 1985). The Institutional Animal Care and Use Committee (IACUC) at Genentech reviewed and approved all animal protocols. To generate *Bmp1* conditional KO mice, *Bmp1*-flox/flox mice^46^ were bred to the *Rosa26*-CreERT2 mouse strain^47^ to produce *Bmp1*-flox/flox;*Rosa26*-Cre-ERT2 mice. *Bmp1*-wt/wt;*Rosa26*-Cre-ERT2 mice were used as controls in this study. Mice from both groups were injected intraperitoneally daily for 5 consecutive days with tamoxifen (Cat# T5648; Sigma) in sunflower oil (S5007-1L; Sigma) at 80 mg/kg. Animals then rest for 2 days and received second dose of tamoxifen again for five consecutive days. Subsequent studies were initiated at least one week after the completion of tamoxifen treatment.

### Bleomycin model of lung fibrosis

The study was carried out in compliance with the ARRIVE guidelines. Adult mice (> 12 weeks) were randomized based on pre-study weights to minimize variance between experimental and control groups. For intratracheal (i.t.) dosing, all mice were lightly anesthetized with isoflurane in an induction chamber. Once anesthetized, the animals were removed from the chamber, manually restrained, the mouth of the animal was opened and the tongue set aside. A 1 ml syringe with 50 µl of sterile injectable isotonic saline or bleomycin (0.72 U/kg (DNC# 0703-3155-01; TEVA) in 50 μl sterile isotonic saline) was connected to a 24 gauge gavage needle. The gavage needle was inserted into the trachea and a dose of either vehicle or bleomycin was delivered intratracheally. After delivery, animals were monitored continuously until fully awake and ambulatory. Mouse whole lungs were harvested at day 24 after bleomycin administration, n = 5 in the saline control group, n = 30 in the bleomycin group in *Bmp1*-cKO and WT control littermates respectively.

### Histology analysis

Formalin-fixed samples of mouse lungs were embedded as a whole and processed to 1 slide per animal, stained with Masson's Trichrome. The extent of pulmonary fibrosis was scored according to the following criteria:

(A) Interstitial fibrosis pattern—number of foci: 0, none detected; 1, ≤ 10; 2, ≤ 15; 3, > 15/all sections, but distinct; 4, multifocally coalescent or locally extensive; 5, diffuse.
(B) Interstitial fibrosis—size of foci: 0, none detected; 1, largest focus ≤ area of ~ 2 alveolar spaces; 2, largest focus ≤ area of ~ 4 alveolar spaces; 3, coalescent (> 4 patent alveolar spaces); 4, locally extensive (60–90% of an entire lobe); 5, diffuse (> 90% of an entire lobe).
(C) Total scores: number of foci × size of foci.

### Hydroxyproline and measurement

Total hydroxyproline was analyzed as previously described^48^ and mass spectrometry and analysis were performed by Metabolic Solutions. Deuterated water labeling was used to assess new collagen synthesis. In brief, mice were injected with deuterated water (DLM-4-99.8-1000; Cambridge Isotope Laboratories) intraperitoneally two weeks prior to the end of the study at 35 mL/kg in 2 divided doses 4 h apart. Afterward, 8% deuterated water in drinking water was provided ad lib in water bottle until the end of the study. Mass spectrometry and analysis were performed by Metabolic Solutions.

### RT-qPCR

Total RNA was purified using RNeasy kit (Qiagen) and treated with DNaseI (LifeTechnologies). Complementary DNA synthesis was carried out with iScript RT Supermix (Bio-Rad). Quantitative PCR was performed in technical triplicates using SYBR Green reagent (Bio-Rad). The relative standard curve method was used for quantitation and expression levels were calculated by normalization to *Hrpt*. The sequences of primers are listed in Supplementary Table 1.

### Western blot

Western blot was carried out in total protein extracts as previously described^49^. Equal amounts of protein lysates were separated by SDS–PAGE, transferred to a nitrocellulose membrane, and subjected to immunoblotting analysis using the following primary antibodies: BMP1 and CICP antibodies were reported previously^34^, Collagen I (1:1000, Abcam, ab21286), GAPDH (1:1000, CST, #5174), β-actin (1:3000, Sigma, a5441). The raw image data are provided in Supplementary Figs. 1–4.

### RNA-seq analysis and plotting

Expression was measured in normalized reads per kilobase per million total reads (nRPKM). RNAseq data on mouse lungs from bleomycin model was published in the NCBI GEO database under accession GSE168529^36^.

### Quantification and statistical analysis

GraphPad Prism was utilized for statistical analysis on Figs. 3, 4, 5 and 6. Statistical details of experiments can be found in figure legends, including the statistical tests used and value and definition of n. Differences were considered to be statistically significant when p < 0.05. Western blot band densitometry was performed using ImageJ software.

## Supplementary Information

Supplementary Information.
