# Supplementary material for: BMP1 is not required for lung fibrosis in mice
Source: Sci Rep. 2022 Mar 31;12:5466. doi: 10.1038/s41598-022-09557-3 (PMC8971496; doi:10.1038/s41598-022-09557-3)
Supplement: Supplementary file 1 — Supplementary Information. [file 41598_2022_9557_MOESM1_ESM.pdf]

BMP1 is not required for lung fibrosis in mice

Hsiao-Yen Ma[1]†, Elsa-Noah N'Diaye[1]†, Patrick Caplazi[2], Zhiyu Huang[3], Alexander Arlantico[3], Surinder Jeet[3], Aaron Wong[3], Hans Brightbill[3], Qingling Li[4], Weng Ruh Wong[4], Wendy Sandoval[4], Lucinda Tam[5], Robert Newman[5], Merone Roose-Girma[5], Ning Ding[1]††

1 Department of Discovery Immunology, Genentech, South San Francisco, CA, USA.

2 Department of Pathology, Genentech, South San Francisco, CA, USA.

3 Department of Translational Immunology, Genentech, South San Francisco, CA, USA.

4 Department of Microchemistry, Proteomics & Lipidomics, Genentech, South San Francisco, CA, USA.

5 Department of Molecular Biology, Genentech, South San Francisco, CA, USA.

† These authors contributed equally.

†† To whom correspondence should be addressed: ding.ning@gene.com

Table 1. mouse primers

Primer	Sequence
Hprt-F	TCAGTCAACGGGGGACATAAA
Hprt-R	GGGGCTGTACTGCTTAACCAG
Bmp1-F	TTGTACGCGAGAACATACAGC
Bmp1-R	CTGAGTCGGGTCCTTTGGC
Tll1-F	GGTTGGTGGTCTCGGGTATTG
Tll1-R	GGCGATGTCACCCCAAAACA
Tll2-F	CCCCTTGCGACCACTCTTG
Tll2-R	CCAGAGCAATATCTCCCCAGAA
Mep1a-F	AATGCTTTGGATACAACCTGCG
Mep1a-R	TCCATCTGCTTGAGGGATCTC
Col1a1-F	GCTCCTCTTAGGGGCCACT
Col1a1-R	CCACGTCTCACCATTGGGG
Col3a1-F	CTGTAACATGGAAACTGGGGAAA
Col3a1-R	CCATAGCTGAACTGAAAACCACC
Serpine1-F	TTCAGCCCTTGCTTGCCTC
Serpine1-R	ACACTTTTACTCCGAAGTCGGT
Timp1-F	GCAACTCGGACCTGGTCATAA
Timp1-R	CGGCCCGTGATGAGAAACT

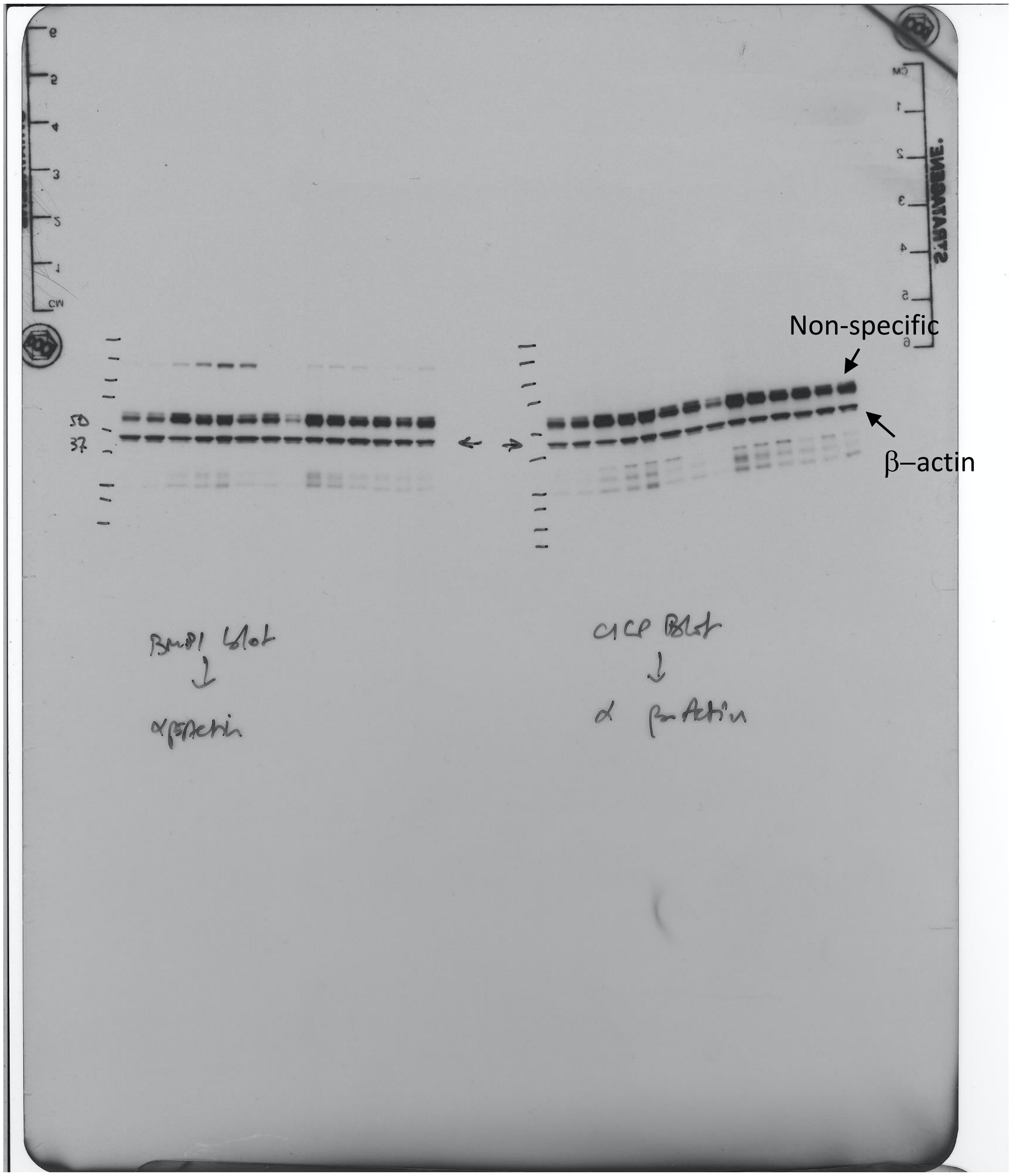

Supplemental Figure 1.
Raw western blot of BMP1 in lung lysates after 24 days bleomycin with β-actin as a loading control.

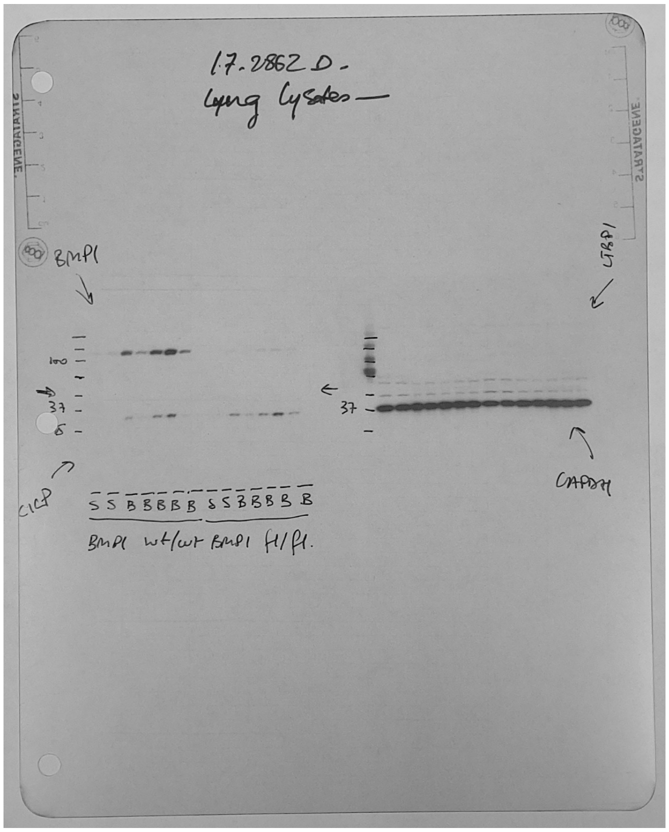

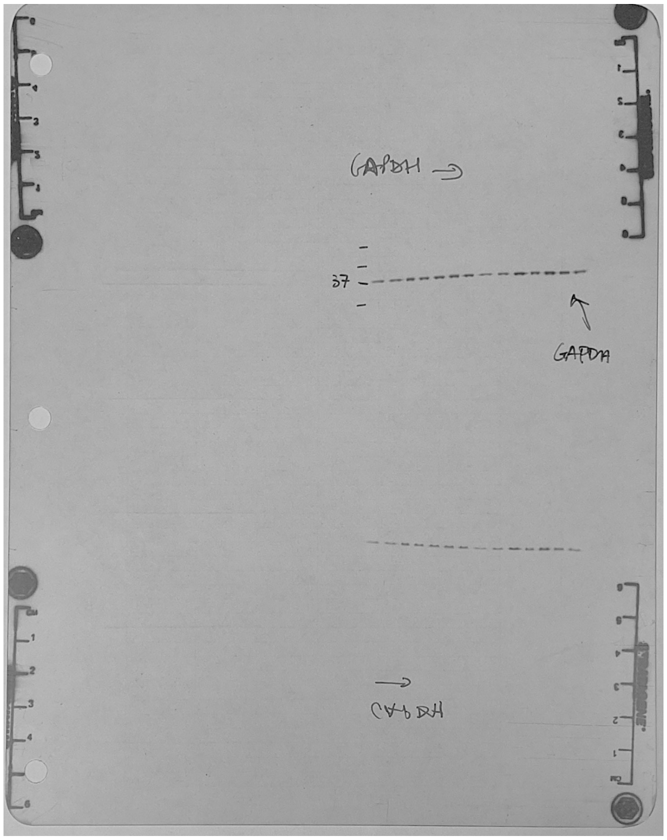

Supplemental Figure 2.
Raw western blot of BMP1 and CICP in lung lysates after 24 days bleomycin with GAPDH as a loading control.

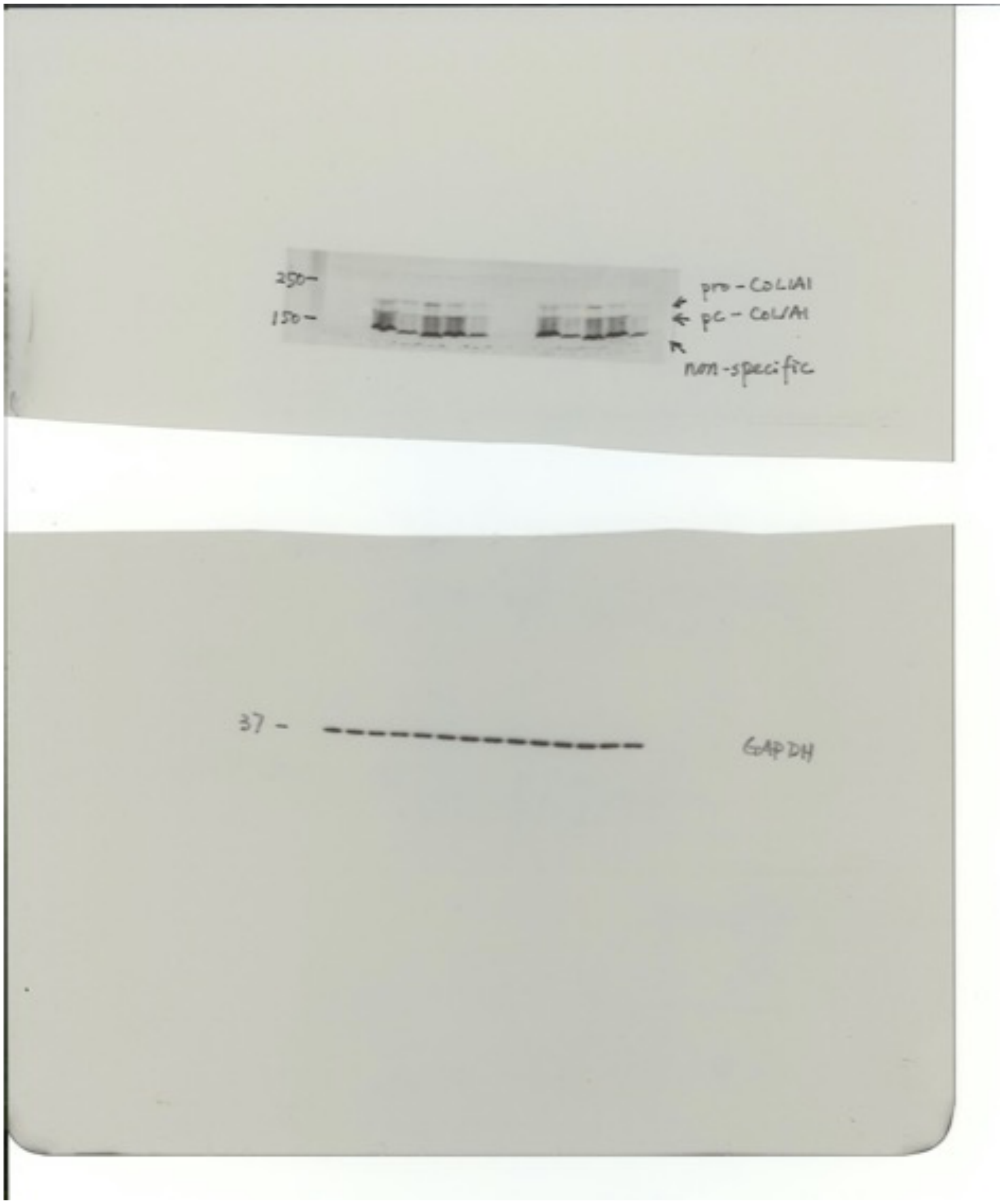

Supplemental Figure 3.
Raw western blot of proα1(I) and pCα1(I) in lung lysates after 24 days bleomycin with GAPDH as a loading control.

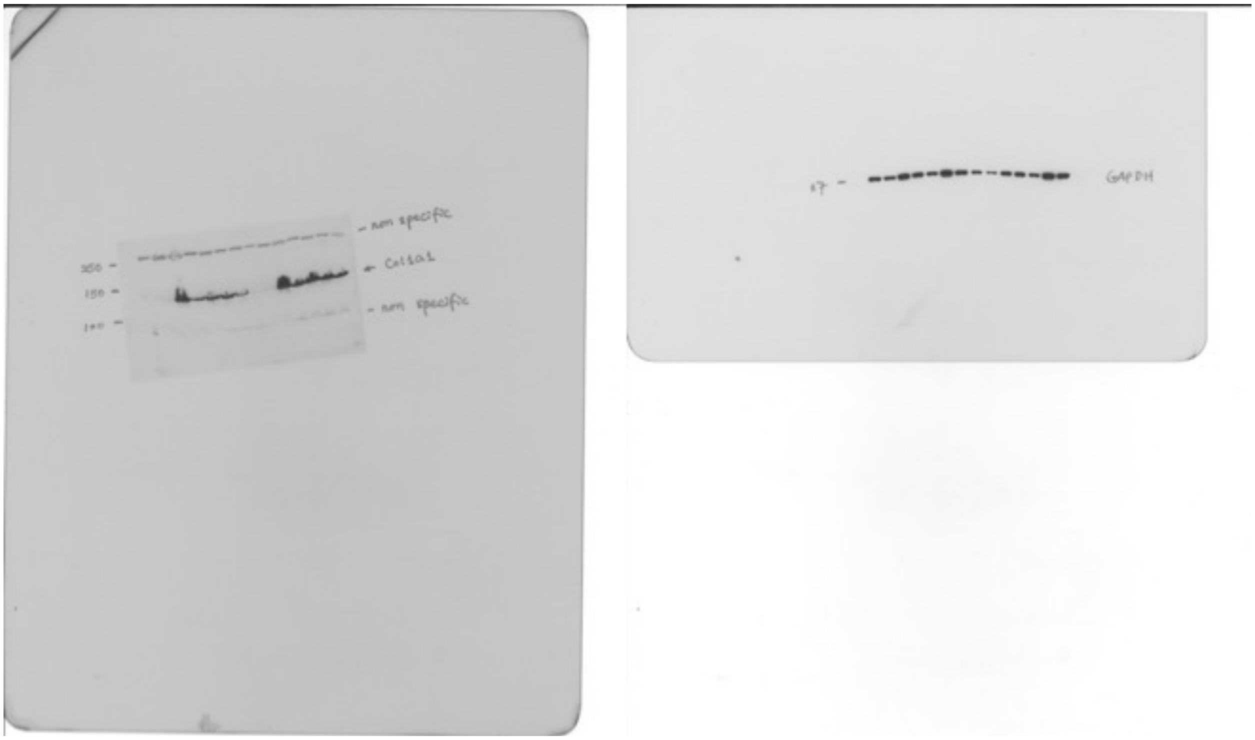

Supplemental Figure 4.
Raw western blot of fully processed COL1 in lung lysates after 24 days bleomycin with GAPDH as a loading control.
